# How does cigarette smoking affect airway remodeling in asthmatics?

**DOI:** 10.18332/tid/156047

**Published:** 2023-01-27

**Authors:** Huihui Lin, Hequan Li

**Affiliations:** 1 Department of Respiratory Diseases, The First Affiliated Hospital of Zhejiang University School of Medicine, Zhejiang University, Zhejiang, China

**Keywords:** asthma, cigarette smoking, airway remodeling, airway inflammation

## Abstract

Asthma is a prevalent chronic airway inflammatory disease involving multiple cells, and the prolonged course of the disease can cause airway remodeling, resulting in irreversible or partial irreversible airflow limitation and persistent airway hyperresponsiveness (AHR) in asthmatics. Therefore, we must ascertain the factors that affect the occurrence and development of airway remodeling in asthmatics. Smokers are not uncommon in asthmatics. However, there is no systematic description of how smoking promotes airway remodeling in asthmatics. This narrative review summarizes the effects of smoking on airway remodeling in asthmatics, and the progress of the methods for evaluating airway remodeling.

## INTRODUCTION

According to statistics, more than 273 million people worldwide have asthma^1^. Asthma will cause recurrent symptoms such as wheezing, shortness of breath, chest tightness, or cough, which often occur at night and/or early in the morning, and most patients can be relieved of the symptoms by themselves or after treatment. At the same time, its severity and attack frequency vary from person to person. Cigarette smoke (CS) has been shown to significantly affect the lungs, including the induction of oxidative stress, inflammatory cell recruitment, and protease/antiprotease imbalance. All these effects exacerbate tissue remodeling and destruction in patients with chronic obstructive pulmonary disease (COPD), resulting in loss of lung function^2^. Although asthma is not as fatal as COPD or other chronic diseases, it can also lead to death in severe cases if it is not well controlled. Controlling asthma is hampered by the presence of airway remodeling. Remarkably, current cigarette smokers are unexpectedly common in adult patients with asthma^3,4^. Therefore, to better control and manage asthma, we must recognize the role of CS in promoting airway remodeling in asthma.

Airway remodeling is widely defined as any change in the structural composition, distribution, thickness, mass, or volume and/or quantity of the airway wall of a patient relative to a healthy person. Asthma can mediate airway remodeling through inflammatory and non-inflammatory reactions in the lungs. On the one hand, airway remodeling can be interpreted as the result of the inflammatory response of the airway to allergens, a process involving a variety of inflammatory cells such as eosinophils, lymphocytes, and mast cells (MCs), among which T lymphocytes play a crucial role. When allergens invade the body, they are endocytosed by antigen-presenting cells [such as dendritic cells (DCs), monocyte-macrophages, etc.], and then T cells, mainly Th2 cells, are activated. Activated CD4+Th2 cells can secrete cytokines such as IL-4, IL-5, and IL-13. In addition to directly activating various inflammatory cells such as MCs, eosinophils, and alveolar macrophages to infiltrate and recruit them in the airways, these cytokines can also aggravate AHR. Moreover, these cytokines can stimulate B lymphocytes to produce immunoglobulin E (IgE). This specific IgE can then immobilize on the surface of MCs, basophils, neutrophils, macrophages, and Natural killer (NK) cells, making them ‘sensitized’. These inflammatory cells, factors, and mediators interact to form a complex network that stimulates mucous cell metaplasia, mucus secretion, fibroblast growth and extracellular matrix protein (ECM) synthesis, airway constriction and induces AHR and airway remodeling^5-9^.In recent years, studies have found that Th17 is also essential in the pathogenesis of asthma and is closely related to the recruitment of airway neutrophils^10^. Meanwhile, regulatory T cells (Tregs) can be recruited to the inflammatory site and release cytokines such as IL-10, which can play anti-regulation^11^. On the other hand, some studies have suggested that airway remodeling in asthma is also the consequence of abnormal damage and repair response of bronchial epithelial cells to the susceptibility of inhaled environmental components. That is, in addition to airway inflammation, epithelial damage directly caused by environmental factors or allergens and the accompanying repair process can promote the occurrence and development of airway remodelling^9,12,13^. Generally speaking, CS will ultimately encourage remodeling airway as long as it can affect every link in the occurrence and development of airway inflammation, damage, and repair in asthma.

Within this narrative review, we discuss the effects of CS on airway remodeling in asthmatics. With regard to airway inflammation, we illustrate three aspects: 1) inflammatory cells; 2) CS-related specific cytokines, such as Thymic Stromal Lymphopoietin (TSLP) and other relevant cytokines; and 3) Receptors for advanced glycation end products (RAGE).

## DEVELOPMENTS

### The effects of CS on airway inflammation in asthmatic patients’ inflammatory cells

Asthma is a heterogeneous group of chronic respiratory diseases. Therefore, to better understand the development of the disease, several asthma phenotypes with unique clinical and inflammatory features have been identified. The current classification of the cellular inflammatory phenotypes of asthma is mainly based on inflammatory cells in the induced sputum. The major categories of asthma phenotypes are eosinophilic, neutrophilic, mixed eosinophilic and neutrophilic, and paucigranulocytic asthma (PGA), which means different types of inflammatory responses in asthmatics^14,15^. Accordingly, the mechanisms leading to airway remodeling may differ for each asthma phenotype. Correspondingly, CS can affect airway remodeling through various mechanisms.

### Eosinophilic asthma

The classical theory holds that activated eosinophils play a vital role in the occurrence and development of asthma. Eosinophils can damage the airway epithelium and accelerate airway remodeling by releasing cationic granule proteins, such as eosinophil cationic protein (ECP), that are highly toxic to the respiratory epithelium^16-18^. Eosinophils are also the primary source of the most potent profibrotic cytokine, TGF-β^13^. Eosinophils are mainly involved in laminin production in epithelial cells, and the MCs are involved in the production of tenascin in fibroblasts. Eosinophils can also act synergistically with MCs as eosinophil products, such as ECP and TGF, interfere with fibroblast growth and matrix protein production. They both promote airway remodeling by increasing the thickness of the laminin and tenascin layers^16,19,20^. Therefore, we can be sure that the damage of the epithelium by activated eosinophils is considered one of the main pathophysiological mechanisms of airway remodeling in asthma. According to the study of Amin et al.^16^, compared with non-smokers, asymptomatic smokers have increased eosinophils in the airway mucosa, and there is a correlation between the number of eosinophils and epithelial integrity in smokers. This finding is consistent with the results within mice constructed by Myung et al.^21^. They found that chronic environmental tobacco smoke exposure increases peribronchial eosinophilic inflammation in chronic ovalbumin-challenged mice. Therefore, we speculate that CS can activate more eosinophils in the airway to recruit more eosinophils and aggravate airway remodeling.

However, some reports show that smokers with asthma do not necessarily have higher eosinophil counts than non-smokers^22,23^. For example, Broekema et al.^24^ reported that smokers were characterized by increased MCs and decreased eosinophil numbers in the bronchial airway walls compared with former smokers and never smokers with asthma. Brüggemann et al.^25^ found that compared with the ovalbumin-sensitized mouse model, the number of eosinophils, IL-4 and IL-13 in the bronchoalveolar lavage fluid (BALF) of ovalbumin combined with CS stimulation-sensitized mouse model decreased.

One of the explanations for these different conclusions is different CS co-exposure schemes. Hence, more experiments are needed to clarify the mechanism through which smoking induces airway remodeling caused by airway inflammation in asthma.

### Neutrophilic asthma

Neutrophilic asthma is rare and mostly more common in critically ill patients^26,27^, and this asthma phenotype generally responds poorly to inhaled corticosteroids (ICSs)^27,28^. So far, a growing number of clinical studies have identified a type of neutrophil-dominant inflammation in the airways of asthmatic patients and advocated that airway neutrophilia is associated with asthma severity^29-31^. In addition to secreting TGF-β, neutrophils can also produce matrix metalloproteinase-9 (MMP-9) and elastase^32-34^, which can negatively affect the airways, including airway narrowing due to airway remodeling, neutrophil elastase-mediated mucus hypersecretion, increased airway smooth muscle responsiveness, and a rapid decline in lung function^35-37^.

Numerous studies pointed out that smokers have significantly higher neutrophil counts than non-smokers^16,38,39^. Airway remodeling should be exacerbated in asthmatics who smoke with elevated neutrophil counts. It is also worth noting that smokers with asthma have features comparable to those in the early stages of COPD compared to non-smokers with asthma, and smokers with asthma have higher neutrophil counts, which play an essential role in the pathophysiology of asthma.

### Mixed eosinophilic and neutrophilic asthma

Most current research on asthma has focused on eosinophils and Th2 inflammation, with little consideration of the potential role of neutrophils and Th1 and Th17 inflammatory pathways. After analyzing the cytokines, chemokines or growth factors, and white blood cell counts in cell-free supernatants obtained from induced sputum in subjects with a wide range of asthma severities, Hastie et al.^40^ argued that airway inflammation in asthma is characterized by overlapping immune pathways. Besides, their results provided examples of Th1, Th2, and Th17 inflammatory mediators interacting with neutrophils and eosinophils and demonstrated overlap in airway inflammation between different T cell types. It is generally believed that Th1/Th2 immune imbalance dominated by the Th2 immune response is one of the critical pathogeneses of asthma. However, the study by Li et al. confirmed that Th2/Th17 responses characterize chronic CS exposure-related asthma. CS can stimulate Th17 cells to secrete more Th17-related cytokines (such as IL-17A, and IL-6), which is significant in asthma^41,42^. IL-17 is involved in neutrophil-dominated inflammation and promotes the accumulation and activation of neutrophils in the lungs, exaggerating inflammatory responses^42,43^. It is noteworthy that although it has not been determined whether Th17 cells can directly promote airway remodeling, the experiment of Zhao et al.^44^ found that the staining of collagen fibers and α-smooth muscle actin decreased. Airway remodeling ameliorated when Th17 cells were absent^44^.

As previously stated, asthmatic patients who smoke have elevated levels of neutrophils, which may be associated with the asthma-COPD overlap syndrome (ACOS)^45,46^. The prevalence of asthma-COPD overlap reported in epidemiological surveys ranges from 9% to 55%, depending on gender and age. However, there are currently different opinions on the mechanisms of overlap, and there is no clear definition and diagnostic criteria for AOCS. Later, GINA/GOLD recommended the revised term ‘asthma-COPD overlap (ACO)’ or ‘asthma+COPD’. ACO is currently primarily defined as persistent airflow limitation with several features commonly associated with asthma and several features commonly associated with COPD^47,48^. Given that COPD is an inflammatory airway disease driven by CS, then in the case of asthma, we can speculate that CS may contribute to asthma having features of COPD or manifesting as ACO, causing further aggravation of airway inflammation^49^. There are indeed reports of high eosinophils and neutrophils in some asthma patients, most of whom have refractory asthma, the lowest lung function, and the highest frequency of daily wheezing^14,50^.

### Paucigranulocytic asthma

The final asthma phenotype is paucigranulocytic asthma (PGA), which is characterized by the absence of evidence of elevated eosinophil or neutrophil numbers in sputum or blood. Furthermore, anti-inflammatory therapy was ineffective in controlling symptoms of this asthma phenotype^51^. In the absence of eosinophils or neutrophils, PGA is considered non-inflammatory or, at best, a low-grade airway inflammatory syndrome associated with airway smooth muscle (ASM) dysfunction and AHR^52,53^.

Airway remodeling may occur in the airways despite the lack of granulocytes, most likely due to macrophages and MCs’ role rather than eosinophils and neutrophils^54-56^. One of the hallmarks of airway inflammation in smokers is an increase in MCs and macrophages in the epidermis and subepithelial layers compared to non-smokers^16^. MCs have emerged as momentous cells in the pathogenesis of asthma and are highly present in asthmatics who smoke^9,25,57^. MCs can mediate epithelial changes by synthesizing and secreting tryptase, chymotrypsin, proinflammatory cytokines such as IL-13 and TNF, and profibrotic cytokines such as TGF-β^58,59^. Macrophages regulate epithelial and stromal cell function by secreting enzymes and inflammatory factors that act directly and indirectly on airway structural cells, such as IL-1β, TNF-α, IL-8, monocyte chemoattractant protein-1(MCP-1), reactive oxygen species (ROS) and MMPs, directly involved in the process of airway remodelling^60,61^.

### TSLP and other relevant cytokines

Firstly, TSLP is an epithelial cell-derived cytokine associated with the pathogenesis of asthma^62^ and belongs to the IL-2 family. The expression of TSLP is increased in the asthmatic airways and correlates with type 2-attractive chemokine expression and disease severity^63,64^. Besides, TSLP has been shown to mediate interactions between airway structural cells and inflammatory cells^65^. When cigarette smoke extracts (CSE) invade the airway combined with an antigen, TSLP located in epithelial cells can induce the expression of OX40 ligand (OX40L) on DCs, and then promote the differentiation of naive CD4+ T cells into Th2 cells, to drive the production of Th2 proinflammatory cytokines, promote airway inflammation through Th2 cell immune response after that. It is worth noting that the pro-inflammatory cytokines produced are high levels of IL-4, IL-5, IL-13, tumor necrosis factor TNF- α, and low levels of IL-10 in this process^66-68^.

On the other hand, Allakhverdi et al.^65^ found that TSLP has a synergistic effect with IL-1 and TNF and in the absence of T lymphocytes and IgE antibodies, may directly activate effector cells of the innate immune system, such as MCs, to accelerate airway remodeling in asthma.

Because of the increase in expression of TSLP, compared with non-smoking asthma patients, smoking asthma patients have significantly increased airway inflammation, and persistent airway inflammation can aggravate airway remodeling through repeated airway epithelial damage and repair.

### Cell membrane receptor RAGE

RAGE is a type of cell membrane receptor highly expressed in alveolar epithelial cells and alveolar macrophages, which can regulate the differentiation of alveolar epithelial cells and affect the development and maintenance of lung structure^69^. The polymorphism of the RAGE gene is associated with the increased incidence of asthma. When allergens stimulate the respiratory system, RAGE can promote the expression of IL-33^5^. In addition to TSLP, the epithelial-derived cytokines IL-33 and IL-25 can also induce the secretion of type 2 cytokines^5,70^. Among them, IL-33 is an effective stimulant of type 2 innate lymphoid cells (ILC2s) and can promote the aggregation of ILCs in the lung. Soon afterwards, ILCs can secrete a mass of Th2 cytokines after activation^71-73^. Studies have demonstrated that RAGE gene knockout and drug inhibition can reduce CS-induced airway inflammation, reducing airway inflammation-related symptoms in patients with asthma. Thus, it can be deduced that RAGE can bind the ligands stimulated by CS and further promote the expression of IL-33, then coordinate a series of inflammatory responses downstream of IL-33 to aggravate the airway damage in patients with asthma, which results in the airway remodeling aggravated finally^5,74,75^.

As mentioned at the beginning of this review, there is a reverse regulatory response in the pathophysiological progress of airway inflammation in asthma, which can gather Tregs to the inflammatory site and release IL-10, a cytokine with regulatory and anti-inflammatory functions, which can weaken the Th2 response in most cases^11^. The results of the Brüggemann et al.^25^ study showed that IL-10 and TGF-βin the OVA+CS exposed group were lower than those in the OVA exposed group. A decrease in the level of IL-10 can lead to a weakening of the inhibitory effect on antigen presentation of macrophages or monocytes, which exacerbates pulmonary inflammation. Although TGF-βis a multipotent cytokine with immunosuppressive effect, no reduction of airway remodeling due to the decrease of TGF-β has been found in this study^25,76,77^.

We should note that the smoking asthmatics mentioned in many current studies may refer to either those who started smoking after the asthma attack in childhood, or those who started smoking before the asthma attack in adulthood. It is known that there is heterogeneity in the clinical presentation of asthma and the type and intensity of airway inflammation and remodeling, implying the importance of identifying the phenotype. However, some specific phenotypes are more common in children, while others are more common in adults^78^. The study of Hancox et al.^79^ found that the relationship between smoking and airflow obstruction differed between asthma phenotypes. Moreover, CS did not cause more severe airflow obstruction in people with childhood-onset asthma that persisted to adulthood than in adults with asthma who smoked. This result suggests that either those with childhood-onset persistent asthma have been less susceptible to the effects of smoking on the airways, or the airway has already been damaged severely by asthma so that further exacerbations become less pronounced^79^. The mechanism of action of CS on airway inflammation and remodeling should then be different for different phenotypes of asthma in children and adults. Nevertheless, there are still few studies on the detailed mechanism, which requires more research to elucidate it.

### The effects of CS on airway damage and repair in asthmatic patients

Except for the smoking-related airway remodeling mechanisms mentioned above, a study has found that smoking can exacerbate airway remodeling by affecting the arginine pathway. Arginase I converts arginine to ornithine, which is the precursor of proline and can gradually convert polyamines through ornithine decarboxylase (ODC). It is worth noting that proline can be used to produce collagen and mucus, and polyamines can promote cell proliferation. Therefore, high arginase activity is likely to promote airway remodeling by increasing collagen deposition and cell proliferation. An experiment found that the immunoreactivity of arginase I and ODC in the smoking group was higher than that in the non-smoking group. Polyamines produced by the arginase I and ODC pathway can aggravate the metaplasia of epithelial cells, increase the proliferation rate of goblet cells, and may increase the area of the submucosal gland in asthmatic patients who smoke. In short, CS can upregulate the expression of arginase I in the airway, causing airway remodelling^45,57,80^.

At present, many reports claim that airway inflammation and remodeling are parallel and interactive factors. Airway remodeling may represent a repair response to inflammation, and we believe that CS can increase the susceptibility of bronchial epithelial cells to inhalation and lead to excessive repair response, which means the occurrence of remodelling^13,81,82^.

For healthy people, there are networks of elastic fibers in the submucosa of their airways that can form discrete longitudinal bundles, and these longitudinal bundles themselves are thickened in asthma. Some studies have found that the elastic fiber networks needed for lung elasticity in smokers are larger than in non-smokers. Therefore, the changes in the size and composition of longitudinal bundles in smoking asthmatics may further aggravate airway remodeling by affecting the mechanical properties of the airway or the folding behavior of the airway mucosa^45,83^. This may be because CSE can significantly promote cell proliferation, and promote ASM to further build and remodel ECM, among which collagen I, collagen III and fibronectin are significantly expressed, thereby promoting airway remodeling^84^. Corresponding to the above, the mouse experiments by Churg et al.^85^ showed that CS could induce the production of pro-fibrotic growth factors in the small airway walls.

There is ample convincing evidence to imply that smokers have more goblet cells and mucus-positive epithelium, increased epithelial thickness, and higher proliferation rates of intact and basal epithelium than non-smoker and ex-smoker asthmatics. In addition, after quitting smoking, asthma control will be better, lung function can be significantly improved, and sputum neutrophils count will decrease, which indicates that the airway changes of asthma caused by smoking could be reversed to some extent through smoking cessation^86,87^.

**Figure 1:**
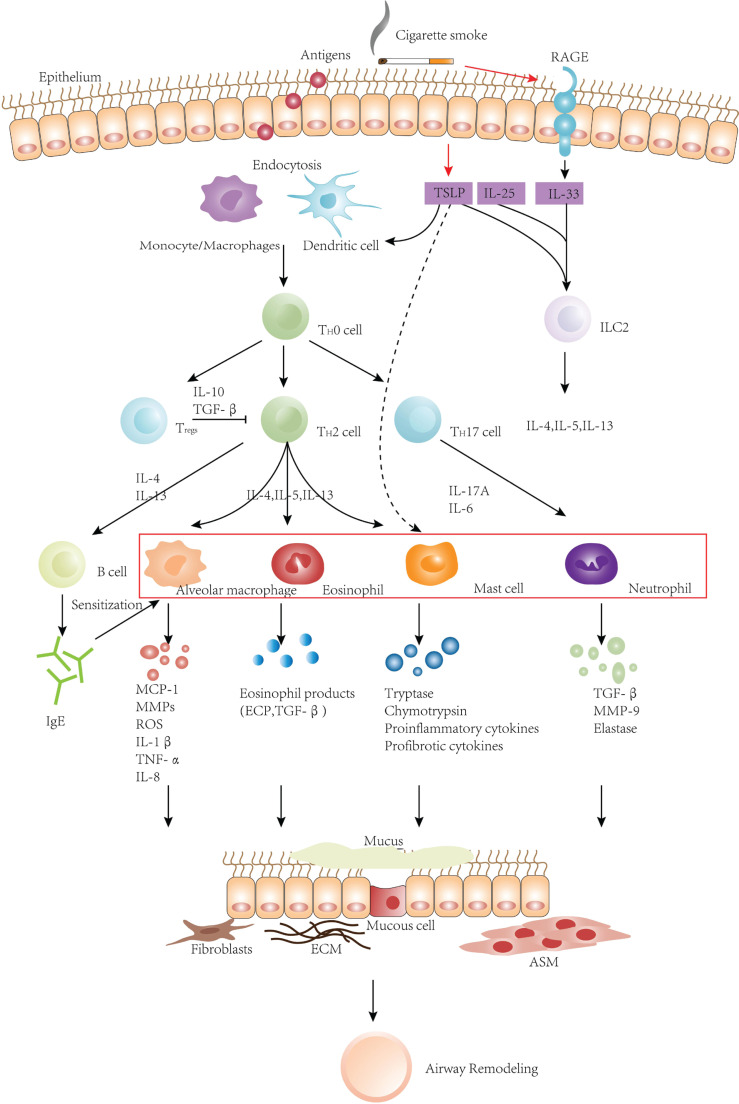
Effects of CSE on airway remodeling in asthma from the perspective of airway inflammation

**Figure 2:**
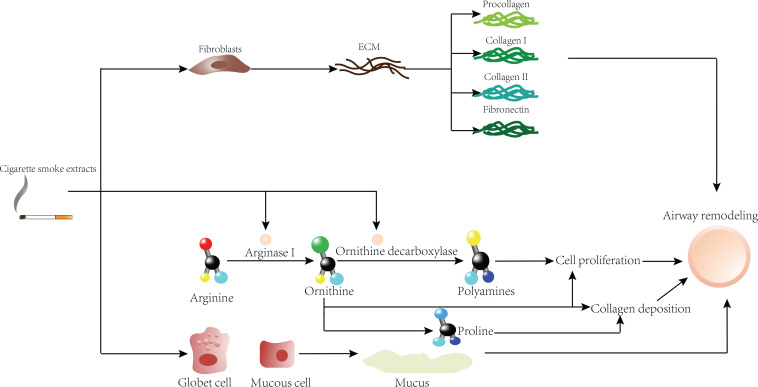
Effects of CSE on airway remodeling in asthma from the perspective of airway damage repair

From many of the above mechanisms (Table 1), we observe that smoking can cause a series of injuries in the airway of asthmatics, and then through inflammatory responses (Figure 1) and/or various damage repair mechanisms (Figure 2) bring about the presence of airway remodeling characterized by more severe irreversible airflow limitation and persistent AHR. After that, the airway will finally show a further decrease in sensitivity to ICSs.

**Table 1:** Differences in airway remodeling mechanisms between smoking and non-smoking asthmatics

			*Non-smoking asthmatic*	*Smoking asthmatic*
**Airway remodeling**	**Inflammatory phenotypes**	**Eosinophilic**	Eosinophils release ECP, TGF, etc.	More eosinophils release more ECP, TGF, etc.*
		**Neutrophilic**	Neutrophils secrete TGF-β, MMP-9 and elastase	More neutrophils secrete more TGF-β, MMP-9 and elastase
		**Mixed eosinophilic and neutrophilic**	Interaction of eosinophils and Th2 immune response	Interaction of neutrophils and Th17 immune response
		**Paucigranulocytic**	MCs and macrophages synthesize and secrete proinflammatory and profibrotic cytokines	More MCs and macrophages synthesize and secrete proinflammatory and profibrotic cytokines
	**Cytokines**		IL-25, IL-33, and TSLP	IL-25 and elevated IL-33, TSLP
	**RAGE**		Bind to the antigens	Bind to the antigens and CS
	**Tregs**		IL-10	Decreased IL-10
**Airway damage and repair**			Mucous cell metaplasia, mucus secretion, fibroblast growth, ECM synthesis and airway constriction	More vigorous mucus cell metaplasia, mucus secretion, fibroblast growth, ECM synthesis, and airway constriction

* There is some disagreement about whether there is an increase in eosinophils, so more research is needed to clarify this.

Allergens are endocytosed by antigen-presenting cells after invading epithelium, and then T cells are activated. Activated T cells can secrete various cytokines, which can directly activate inflammatory cells such as MCs, eosinophils, and alveolar macrophages to infiltrate and recruit them in the airways. Besides, these cytokines can stimulate B lymphocytes to produce IgE to sensitize MCs, basophils, neutrophils, macrophages, and NK cells. These inflammatory cells interact with inflammatory factors and eventually lead to airway remodeling. TSLP, IL-33, and IL-25 are essential factors to activate the pulmonary inflammatory response, and they can all stimulate the secretion of type 2 cytokines. TSLP might directly activate effector cells of the innate immune system, such as MCs. In addition to stimulating the increase of eosinophils, neutrophils, mast cells, and macrophages (red box), CS can also combine with RAGE to further stimulate the expression of IL-33, gradually aggravating airway remodeling.

First, CSE can directly act on fibroblasts to further produce collagen I, collagen III and fibronectin, which are deposited on the airway wall and increase the thickness of the airway wall. Second, CSE can further exacerbate airway remodeling by affecting the arginine pathway. In addition, goblet cells and mucus-positive epithelial cells increased under CSE stimulation, and mucus secretion is increased, resulting in increased epithelial thickness.

### Evaluation methods of airway remodeling

ICSs are the first-line treatment for asthma^47^, reducing asthma symptoms, improving lung function and quality of life, and reducing airway inflammation and AHR. In spite of this, these beneficial effects of ICSs have been reported to be diminished in asthmatics who smoke, and active smoking impairs the efficacy of short-term oral corticosteroids for chronic asthma. Tamimi holds the view that this occurs because CS induces corticosteroid resistance. The primary mechanism by which this resistance arises is reduction of the histone deacetylase 2 (HDAC2) enzyme system^88-91^. Therefore, it is reasonable to conclude that CS-induced exacerbation of airway remodeling in asthma patients is not well relieved by ICSs.

To better manage asthma, it is essential to establish a reliable system to assess airway remodeling. Firstly, histological examination is vital. Initially, light, fluorescence, or electron microscopy can directly evaluate airway remodeling by histological analysis of autopsied or resected lungs. Later, the bronchoscopic mucosal biopsy is widely used to assess airway remodeling directly. However, the size and depth of the specimen obtained by this method are limited. Afterwards, some studies began to use the reflection of pulmonary function to airflow limitation as an indirect representation of airway remodeling. Nevertheless, this approach may be influenced by diseases or short-term asthma control^92^.

In recent years, the concept of biomarkers has been widely applied in all kinds of medical fields. At present, biomarkers of airway remodeling in asthma can be obtained from various sources such as urine, blood, bronchoalveolar lavage fluid, induced sputum, exhaled air agglutination, bronchial biopsy, and some scholars even proposed that imaging technologies can also act as markers^93^. Several studies from BALF and biopsy specimens have suggested viable biomarkers such as MMP-9, protease-activated receptor 2, and even its ligands associated with airway remodeling^94,95^. Nevertheless, these biomarkers are acquired by invasive procedures, so some biomarkers from non-invasive methods are necessary. Experimental studies have demonstrated that galectin-3 (Gal-3) in serum can effectively reflect the reduction of bronchial smooth muscle (BSM), eosinophilic inflammation, and muscle protein components in asthmatics, but this requires more experiments to verify^96-98^. Like Gal-3, chitinase-3-like protein 1 (CHI3L1), also known as YKL-40, can partially reveal changes in airway structure, reflecting airway remodeling. Chupp et al.^99^ found that YKL-40 levels in serum correlated with the thickness of the airway subepithelial basement membrane. However, these biomarkers can also be elevated in other fibrotic diseases, so some results from the respiratory tract rather than serum are more valuable for showing airway remodeling. Another biomarker worth mentioning is periostin. Periostin, an indispensable regulator of the ECM, is highly expressed in the lungs of asthmatics and can interact with other mediators in the sub-epithelial space to promote ASM remodeling and sub-epithelial fibrosis. Periostin also promotes eosinophil recruitment and mucus secretion. Therefore, periostin is a marker of asthma disease progression^100-102^. In conclusion, finding reliable airway remodeling biomarkers to assist in classifying asthma phenotypes and further combining multiple phenotypes for precise treatment has become one of the development directions of asthma treatment.

Another method refers to imaging studies. Although computed tomography (CT) cannot reflect pathological findings, it can show a wide range of airway/lung morphology and quantitatively evaluate the airway size to obtain the lumen diameter, wall thickness, total airway area, and lumen area in cross-section^103^.

A joint working group of the American Thoracic Society (ATS) and the European Respiratory Society (ERS) conducted a rigorous review of the most advanced stereological methods in lung morphometry. It issued an official research policy statement in 2010. This document sets specific standards to promote the comparability of morphometric studies in lung research, which is a new starting point for research on airway remodeling. We recognize that quantitative methods of evaluating airway remodeling must include the direction of the 2D cross-section relative to the longitudinal axis of the airway and realize that the airway’s position significantly influences the quantitative parameters analysed^104^. Later, Hartley et al.^105^ applied high-resolution imaging, namely quantitative computed tomographic (QCT), to the radiological analysis of airway morphology, reflecting airway remodeling in patients with asthma or COPD by OCT parameters.

**Figure 3:**
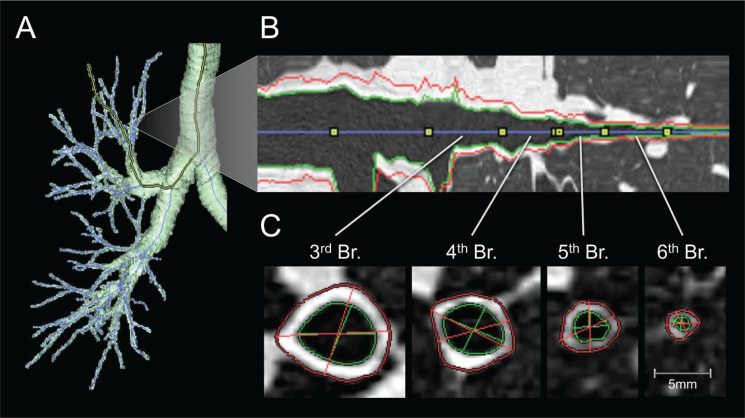
Software assessment of airway remodeling

Factor analysis of QCT parameters in asthmatic patients and patients with COPD combined determined three components, with proximal airway percentage wall area (%WA), air trapping, and Perc15 (Hounsfield units below which 15% of the voxels lie) values, which can reflect airway remodeling in patients with asthma or COPD to some extent^105^. Aysola et al.^106^ found that the airway wall of patients with severe asthma was thicker than that of patients with mild or non-asthma by multidetector computed tomography (MDCT). They also found that the thickening of the airway wall was positively correlated with the pathological remodeling index and the degree of airflow obstruction. Berair et al.^107^ also used QCT to quantitatively measure the morphology and density of proximal airway remodeling and air retention to analyze the relationship between QCT and airway remodeling in bronchial biopsy specimens of patients with asthma. They observed that the proximal airway morphometry obtained by QCT was closely related to epithelial thickness and airway smooth muscle, which means that airway remodeling can be evaluated to some extent by QCT. Likewise, Patyk et al.^108^ used QCT to observe 83 patients with long-term asthma and found that most of the patients with long-term asthma had airway remodeling, which was mainly related to medium and small airways, indicating that imaging techniques such as QCT help evaluate airway wall thickness in patients with asthma. There is much mature software at home and abroad in assessing airway remodeling (Figure 3), which has been employed in clinical research^109^.

## CONCLUSION

CS can further aggravate airway remodeling in asthmatics, either by affecting airway inflammation or the mechanism of airway damage repair. Therefore, quitting smoking is urgent for patients with asthma, while quitting smoking can improve asthma control. At the same time, the evaluation methods of airway remodeling are updated year by year, and a more accurate assessment of airway remodeling will help us have a more precise grasp of the pathophysiological process of asthma. One of the main reasons for the poor control of asthma is that smokers are not uncommon in patients with asthma. CS will aggravate airway remodeling in patients with asthma and the existence of more severe airway remodeling reduces airway sensitivity to ICSs. Thus, quitting smoking is necessary for asthma, and tobacco management is hence essential for asthma management.

## Data Availability

Data sharing is not applicable to this article as no new data were created.
